# Aim18p and Aim46p are chalcone isomerase domain–containing mitochondrial hemoproteins in *Saccharomyces cerevisiae*

**DOI:** 10.1016/j.jbc.2023.102981

**Published:** 2023-02-04

**Authors:** Jonathan M. Schmitz, John F. Wolters, Nathan H. Murray, Rachel M. Guerra, Craig A. Bingman, Chris Todd Hittinger, David J. Pagliarini

**Affiliations:** 1 Department of Biochemistry, University of Wisconsin–Madison, Madison, Wisconsin, USA; 2 Morgridge Institute for Research, Madison, Wisconsin, USA; 3 Laboratory of Genetics, Center for Genomic Science Innovation, Wisconsin Energy Institute, J.F. Crow Institute for the Study of Evolution, University of Wisconsin–Madison, Madison, Wisconsin, USA; 4 DOE Great Lakes Bioenergy Research Center, University of Wisconsin–Madison, Madison, Wisconsin, USA; 5 Department of Cell Biology and Physiology, Washington University School of Medicine, St Louis, Missouri, USA; 6 Department of Biochemistry and Molecular Biophysics, Washington University School of Medicine, St Louis, Missouri, USA; 7 Department of Genetics, Washington University School of Medicine, St Louis, Missouri, USA

**Keywords:** mitochondria, hemoprotein, chalcone isomerase, yeast, uncharacterized protein, AIM, Altered Inheritance of Mitochondria, BME, β-mercaptoethanol, CHI, chalcone isomerase, CoQ, coenzyme Q or ubiquinone, DMQ, demethoxyubiquinone, IMM, inner mitochondrial membrane, LDS, lithium dodecyl sulfate, NFDM, nonfat dry milk, PDB, Protein Data Bank, PPHB, polyprenyl-4-hydroxybenzoate, SC, synthetic complete, SEC, size-exclusion chromatography, TBST, tris buffered saline plus 0.05% tween-20 (v/v), TCA, trichloroacetic acid, TCEP, tris(2-carboxyethyl)phosphine, (v/v), percent volume per 100 ml volume, (w/v), percent weight per 100 ml volume

## Abstract

Chalcone isomerases (CHIs) have well-established roles in the biosynthesis of plant flavonoid metabolites. *Saccharomyces cerevisiae* possesses two predicted CHI-like proteins, Aim18p (encoded by YHR198C) and Aim46p (YHR199C), but it lacks other enzymes of the flavonoid pathway, suggesting that Aim18p and Aim46p employ the CHI fold for distinct purposes. Here, we demonstrate using proteinase K protection assays, sodium carbonate extractions, and crystallography that Aim18p and Aim46p reside on the mitochondrial inner membrane and adopt CHI folds, but they lack select active site residues and possess an extra fungal-specific loop. Consistent with these differences, Aim18p and Aim46p lack CHI activity and also the fatty acid–binding capabilities of other CHI-like proteins, but instead bind heme. We further show that diverse fungal homologs also bind heme and that Aim18p and Aim46p possess structural homology to a bacterial hemoprotein. Collectively, our work reveals a distinct function and cellular localization for two CHI-like proteins, introduces a new variation of a hemoprotein fold, and suggests that ancestral CHI-like proteins were hemoproteins.

Chalcone isomerases (CHIs) catalyze one of the essential steps in plant flavonoid biosynthesis (1, 2, 3). In the CHI reaction, bicyclic chalcones are isomerized into tricyclic flavanones (2, 4). This reaction is initiated by a catalytic arginine and supported through hydrogen bonding with other active site residues. Mutations to any of these residues reduce catalytic activity significantly (5).

Recently, CHIs were shown to belong to a larger family of CHI domain–containing proteins that also contains a clade of noncatalytic fatty acid–binding proteins (6). Additionally, a recent study of reconstructed ancestral CHI domain–containing proteins suggested that this protein family evolved from a noncatalytic ancestor (7), raising the possibility that there may be additional atypical CHI domain–containing protein functions yet to be discovered.

Almost 20 years ago, Gensheimer and Musheigan discovered that the CHI domain–containing protein family extends into fungi, slime molds, and γ-proteobacteria (8). Previous studies mention the presence of a fungal CHI domain–containing protein family (6, 9, 10, 11, 12, 13) and speculate whether these proteins have CHI activity; however, to date, no functional characterization of the fungal CHI domain–containing proteins has been reported.

*Saccharomyces cerevisiae* contains two paralogous CHI domain–containing proteins, Aim18p (encoded by YHR198C) and Aim46p (encoded by YHR199C). More than a decade ago, a high-throughput screen in *S. cerevisiae* identified 100 genes whose absence disrupts the inheritance of mitochondrial DNA by daughter cells. Aim18p and Aim46p exceeded the threshold for this study and therefore were assigned the standard name Altered Inheritance of Mitochondria (*AIM*). To our knowledge, no study has explicitly explored the molecular function of Aim18p and Aim46p in *S. cerevisiae*.

In this study, we find that Aim18p and Aim46p adopt the canonical CHI fold with an additional loop specific to fungi. We also show that Aim18p and Aim46p lack both CHI activity and fatty acid–binding activity *in vitro*. Finally, we demonstrate that Aim18p and Aim46p are hemoproteins and that this heme-binding property is conserved widely among diverse fungi. Our findings expand the noncatalytic ancestor hypothesis by adding heme-binding to the list of functions of CHI domain–containing proteins.

## Results

### Aim18p and Aim46p are yeast proteins with sequence homology to plant CHIs

**Figure 1:**
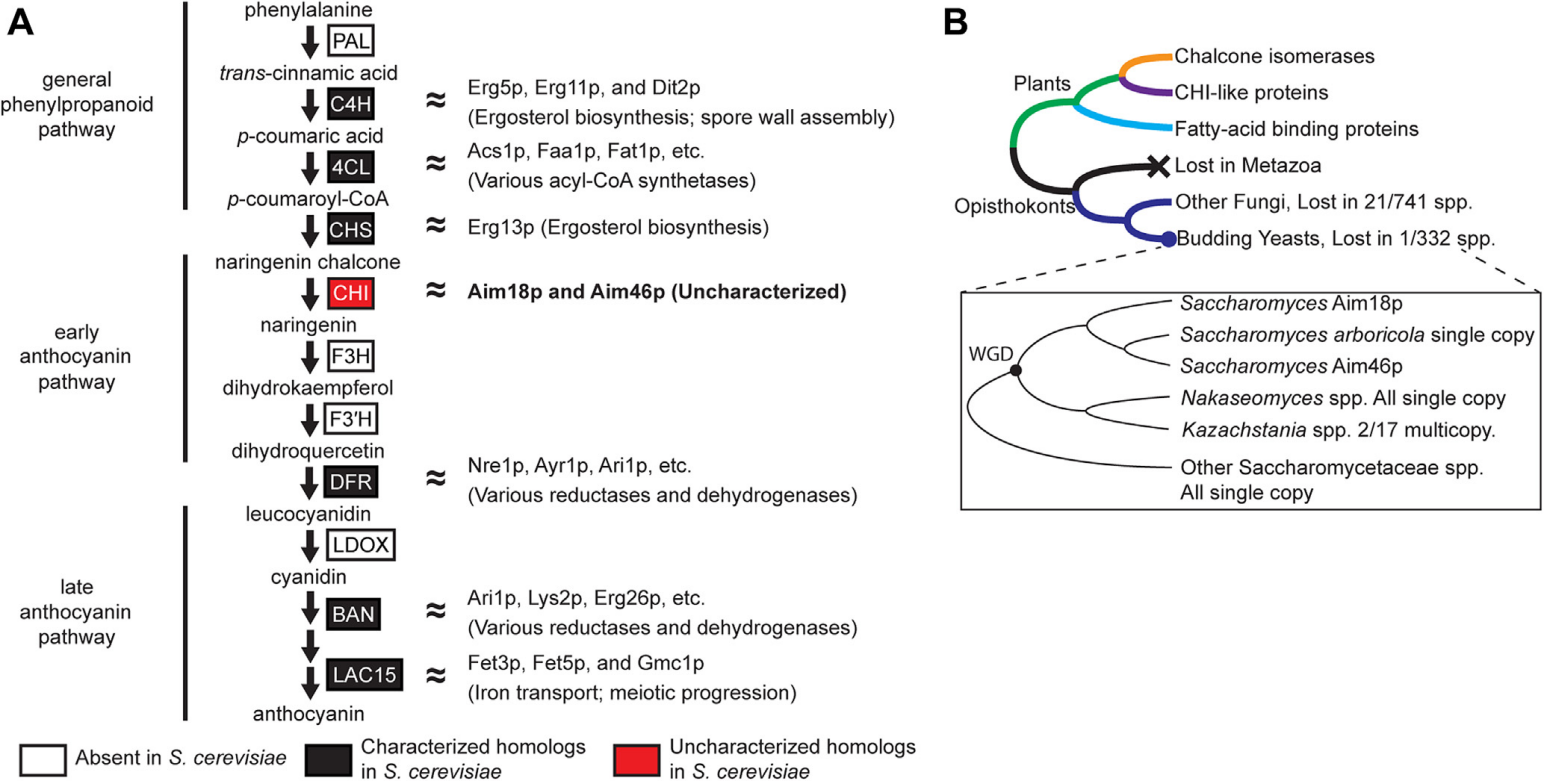
**Aim18p and Aim46p are yeast proteins with sequence homology to plant CHIs.***A*, general flavonoid biosynthetic pathway with key enzymes and intermediates detailed. DELTA-BLAST searches for plant flavonoid biosynthetic pathway members were performed against the *Saccharomyces cerevisiae* genome, detailed in Table S1. *White boxes* indicate that *S. cerevisiae* homologs of plant flavonoid biosynthetic proteins are absent. *Black boxes* indicate that distant *S. cerevisiae* homologs of plant flavonoid biosynthetic proteins are characterized proteins; protein names and functions are noted at *right*. The *red box* indicates that *S. cerevisiae* homologs of the plant flavonoid biosynthetic protein are uncharacterized proteins. *B*, simplified phylogenetic representation of CHI domain–containing proteins across plants and fungi (Fig. S1*A*). Magnified region in the box highlights species descended from the yeast whole genome duplication (WGD); note that no extant species retain the second copy from the WGD, and *AIM18* and *AIM46* are present in tandem in *Saccharomyces* spp., except for *Schefflera arboricola.* CHI, chalcone isomerase.

We sought to investigate whether *S. cerevisiae* possesses a flavonoid biosynthesis pathway by searching for homologs of plant flavonoid biosynthetic enzymes (14). We found distant homologs for multiple enzymes, but *S. cerevisiae* lacked obvious candidates for four pathway enzymes (Fig. 1*A*). Additionally, most of the *S. cerevisiae* homologs are known either to bind notably different substrates than their plant counterparts or to perform entirely different biochemical reactions. For example, the *S. cerevisiae* homolog of chalcone synthase is Erg13p, an HMG-CoA synthase involved in ergosterol biosynthesis, not the biosynthesis of naringenin chalcone, the natural substrate for CHIs (Fig. 1*A*). Thus, while there are some distantly related, sequence-level homologs of some flavonoid biosynthesis genes (Table S1), this pathway most likely lacks functional conservation in *S. cerevisiae*.

In contrast to the other flavonoid biosynthesis pathway homologs in *S. cerevisiae*, the CHI homologs Aim18p and Aim46p remain uncharacterized. Since *S. cerevisiae* does not have a functionally conserved chalcone synthase and likely lacks a functional flavonoid biosynthesis pathway, Aim18p and Aim46p probably do not perform the canonical CHI reaction.

**Figure S1:**
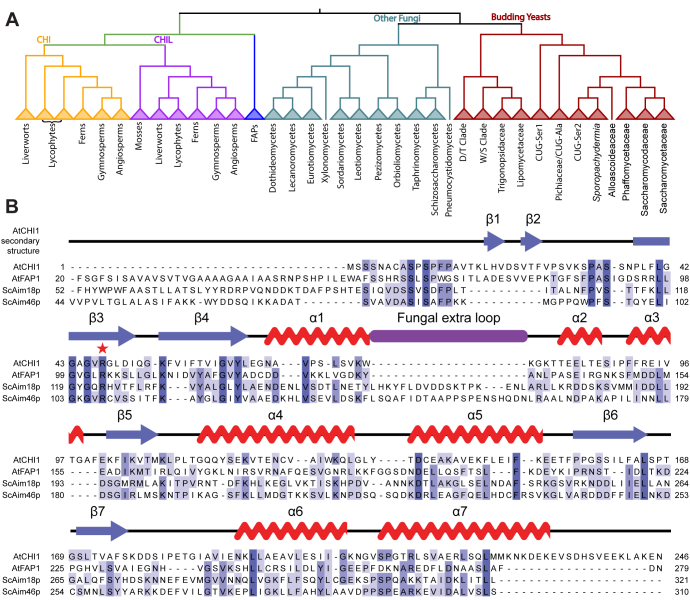


Homologs of *S. cerevisiae* Aim18p and Aim46p are broadly distributed across the fungal kingdom. To interrogate the phylogenetic relationship between fungal and plant CHIs, we combined CHI plant protein sequences (7) and CHI homologs from over 1000 sequenced fungal genomes (15, 16), and we expanded the CHI domain–containing protein family tree (6) (Figs. 1*B* and S1*A*). All fungal CHI domain–containing proteins are an outgroup to the plant CHI domain–containing proteins. Aim18p and Aim46p are encoded by paralogs that likely arose from a tandem gene duplication event specifically in the lineage leading to the genus *Saccharomyces.* The single copy present in the published *Saccharomyces arboricola* genome likely resulted from a fusion of these paralogs, as evidenced by different phylogenetic affinities between its 5′ and 3′ ends (see Experimental procedures). The proteins have significantly diverged in sequence, but both retain a clear CHI domain. Protein sequence alignments of AtCHI1, AtFAP1, Aim18p, and Aim46p demonstrate that Aim18p and Aim46p have sequence similarity in the more structured regions of previously crystallized CHI domain–containing proteins (2, 6), especially the β3 [LXGXGXR] motif that houses the catalytic arginine (Fig. S1*B*).

### Aim18p and Aim46p are inner mitochondrial membrane proteins

**Figure 2:**
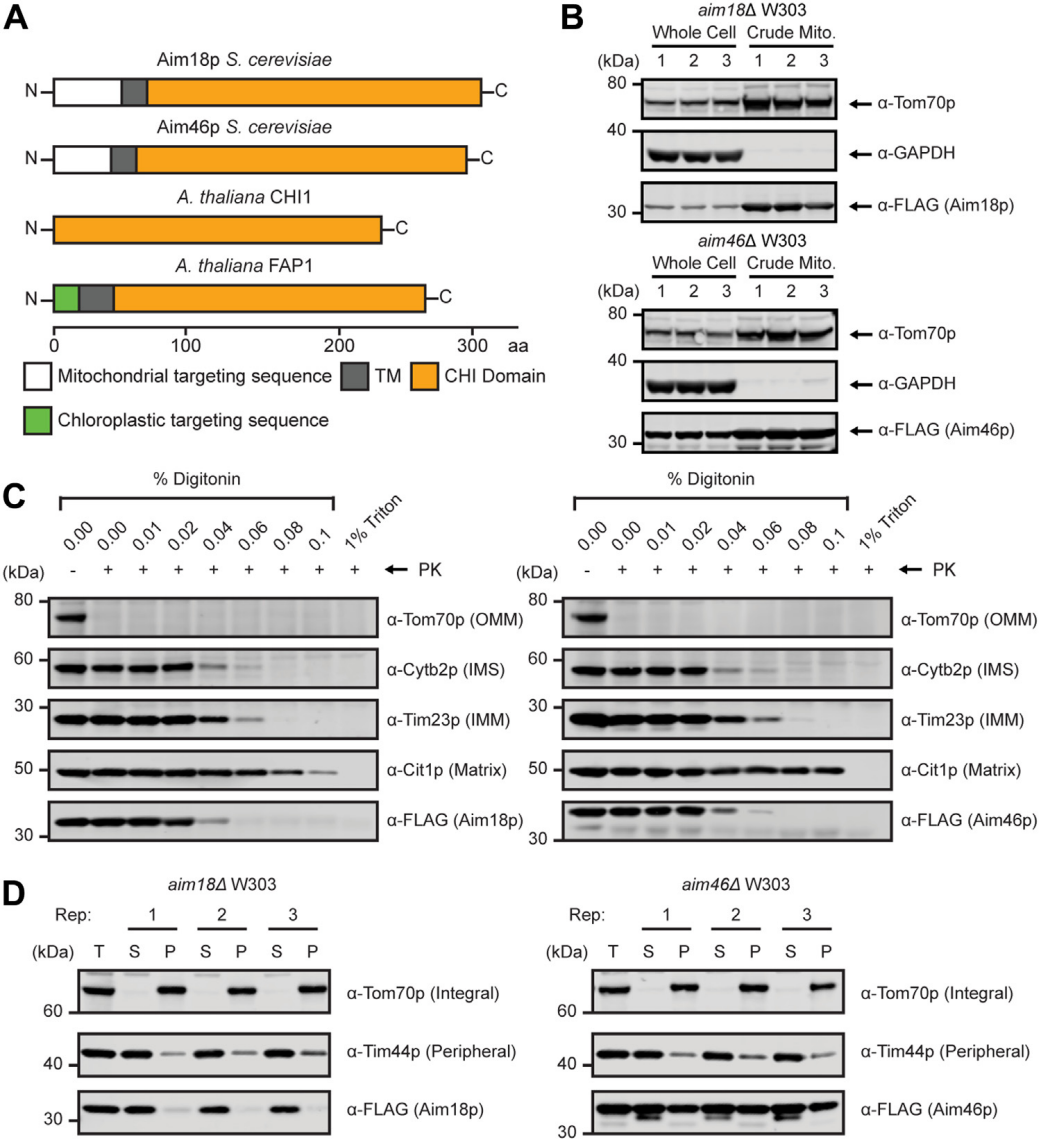
**Aim18p and Aim46p are inner mitochondrial membrane proteins.***A*, cartoon diagram of Aim18p, Aim46p, AtCHI1, and AtFAP1 protein sequences. Organellar targeting sequences, transmembrane domains, and CHI domains are highlighted with their corresponding amino acid residue number. *B*, western blot of Aim18p and Aim46p mitochondrial purifications compared to whole cells. Tom70p, mitochondrial; GAPDH, cytoplasmic; Aim18p-FLAG and Aim46p-FLAG, mitochondrial. *n* = 3 independent biological replicates. *C*, proteinase K protection assay of yeast crude mitochondria expressing Aim18p-FLAG and Aim46p-FLAG. Yeast crude mitochondria were treated with 300 μg proteinase K in the presence of increasing concentrations of detergent. 1% Triton was a control for full mitochondrial solubilization. Representative blot from two independent experiments. *D*, sodium carbonate extraction of yeast crude mitochondria expressing Aim18p-FLAG and Aim46p-FLAG. Yeast crude mitochondria were treated with 100 μl of 100 μM sodium carbonate for 30 min on ice. *n* = 3 independent technical replicates. CHI, chalcone isomerase; IMM, inner mitochondrial membrane; IMS, intermembrane space; OMM, outer mitochondrial membrane.

**Figure S2:**
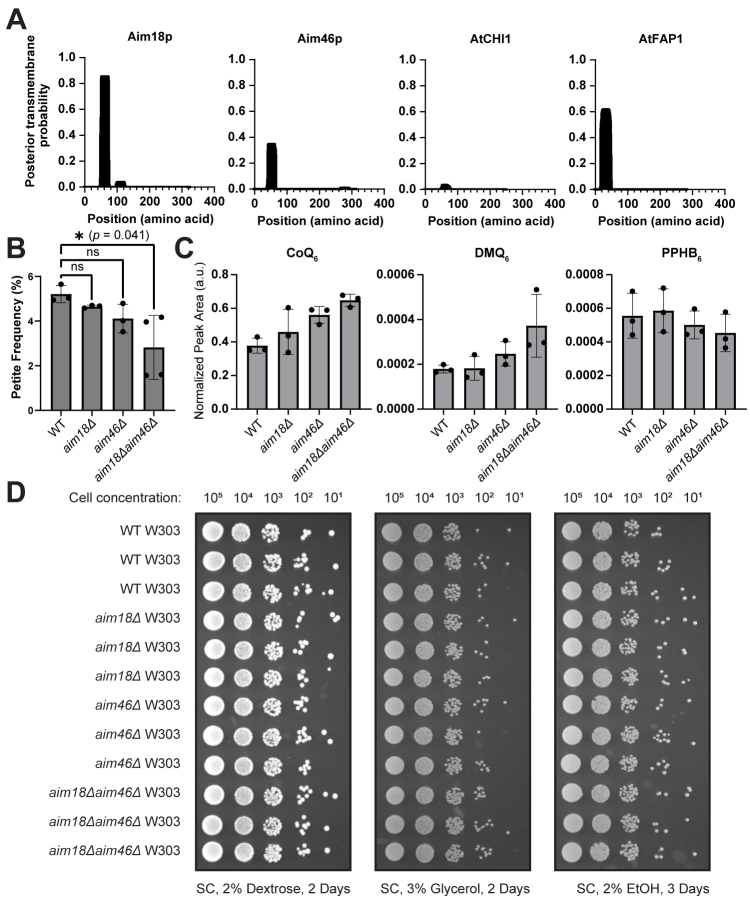


Aim18p and Aim46p each contain N-terminal mitochondrial targeting sequences (Fig. 2*A*), as predicted by MitoFates (17), and an approximately 18 amino acid residue N-terminal transmembrane domain (Fig. 2*A*), as predicted by Phobius (18) (Fig. S2*A*). Crude mitochondrial purifications showed a strong mitochondrial enrichment for Aim18p-FLAG and Aim46p-FLAG compared to whole cell fractions (Fig. 2*B*), which is consistent with previous high-throughput studies that localized these proteins to mitochondria (19, 20, 21, 22). The submitochondrial localization of Aim18p and Aim46p is ambiguous; early studies placed these proteins on the outer mitochondrial membrane (23), while more recent studies suggest they reside on the inner mitochondrial membrane (IMM) (21, 22).

To clarify the localization of these proteins, we performed proteinase K protection assays on crude mitochondrial purifications expressing FLAG-tagged variants of Aim18p and Aim46p. Immunoblots for various mitochondrial markers and our FLAG-tagged target proteins suggest that Aim18p and Aim46p are localized to the IMM (Fig. 2*C*). A recent high-throughput study suggested that Aim18p and Aim46p might be peripherally associated with a membrane *via* their transmembrane domains (21). To determine whether Aim18p′s and Aim46p′s predicted hydrophobic regions behave as transmembrane domains or alternatively associate peripherally with the membrane in a manner similar to amphipathic helices, we performed sodium carbonate extractions on the same crude mitochondrial purifications used in Figure 2*C* (24). Upon treatment with sodium carbonate, Aim18p was liberated into the soluble fraction, while a significant proportion of Aim46p remained in the insoluble fraction (Fig. 2*D*), suggesting that Aim46p′s transmembrane domain might be more strongly associated with the IMM.

*AIM18* and *AIM46* were previously implicated in mitochondrial inheritance in a high-throughput genetic screen (25), but we were unable to repeat this phenotype (Fig. S2*B*). Aim18p also exhibited correlated expression patterns with enzymes involved in coenzyme Q (CoQ) biosynthesis (26), but we did not observe altered CoQ levels in *aim18* and/or *aim46* deletion mutants (Fig. S2*C*). We also did not observe any respiratory deficiency or growth phenotype in these strains (Fig. S2*D*), suggesting that Aim18p and Aim46p are not required for mitochondrial respiration.

### Aim18p and Aim46p adopt the CHI-fold but lack CHI-fold activities

**Figure 3:**
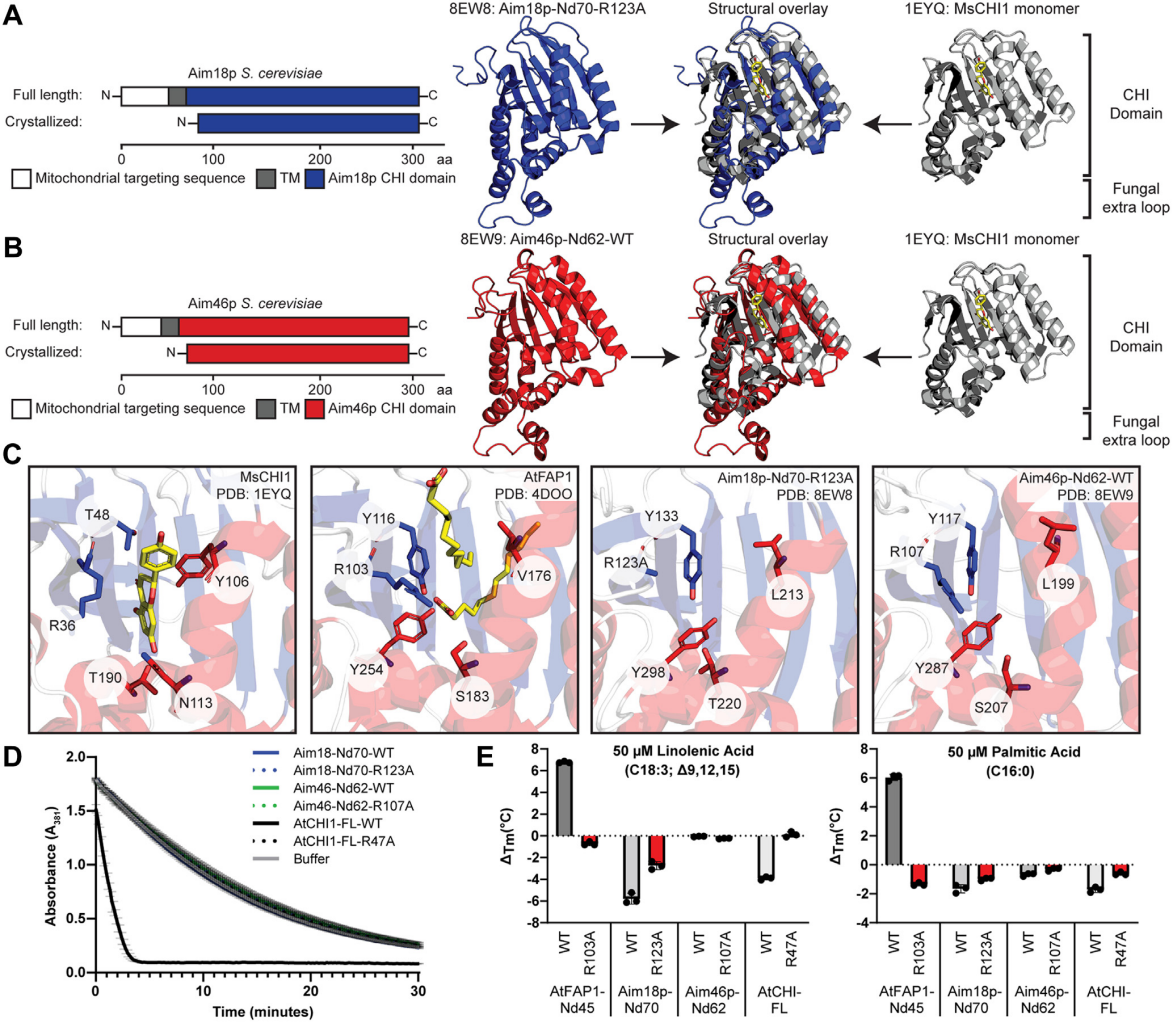
**Aim18p and Aim46p adopt the CHI fold but lack CHI-fold activities.***A*, diagram of crystallized, truncated Aim18p-Nd70-R123A (*blue*) and structural overlay with CHI from *Medicago sativa* (*gray*, PDB: 1YEQ). *M. sativa* CHI naringenin chalcone ligand highlighted in *yellow*. *B*, cartoon of crystallized, truncated Aim46p-Nd62-WT (*red*) and structural overlay with CHI from *M. sativa* (*gray*, PDB: 1YEQ). *M. sativa* CHI naringenin chalcone ligand highlighted in *yellow*. *C*, active site comparisons between MsCHI1, AtFAP1, Aim18p, and Aim46p. Bound ligands for MsCHI1 (naringenin) and AtFAP1 (laurate) highlighted in *yellow*. Alpha helices colored in *red*, beta sheets colored in *blue*, and linker regions colored in *gray*. *D*, chalcone isomerase assay with 10 nM CHI domain–containing proteins and 200 μM naringenin chalcone. Conversion of naringenin chalcone to naringenin measured by A_381_ readings taken every 10 s for 30 min (mean ± SD, *n* = 3 independent technical replicates). Note that the activity traces for all protein constructs except for AtCHI1-FL-WT are indistinguishable due to their overlap with the buffer negative control. *E*, thermal shift response of 5 μM CHI family proteins in response to a 1-h incubation with 50 μM fatty acids compared to a DMSO control (mean ± SD, *n* = 3 independent technical replicates). CHI, chalcone isomerase.

**Figure S3:**
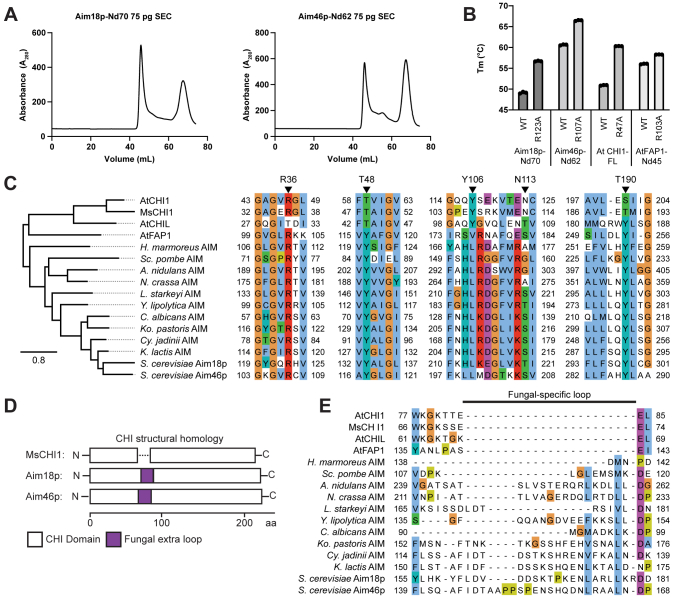


In the absence of a growth phenotype, we employed biochemical techniques to explore the function of Aim18p and Aim46p. Full-length constructs of Aim18p and Aim46p possessing N-terminal 8X-His tags were insoluble, but truncating the N-terminal mitochondrial targeting sequence and transmembrane domain for Aim18p and Aim46p (Aim18p-Nd70 and Aim46p-Nd62, respectively) dramatically increased solubility (Fig. 3, *A* and *B*). These recombinant proteins were expressed in *Escherichia coli* and purified to high concentrations (>20 mg/ml in several instances) and were amenable to size-exclusion chromatography (SEC). Both Aim18p and Aim46p had elution volumes similar to ∼30 kDa proteins (See Experimental procedures; Fig. S3*A*), which suggests that they exist as monomers. We also generated point mutations of the CHI catalytic arginine—Aim18p-Nd70-R123A and Aim46p-Nd62-R107A—to serve as experimental controls. Each protein exhibited robust thermal melt temperatures ranging from ∼48 to 67 °C, indicating stably folded protein (Fig. S3*B*). WT Aim46-Nd62 readily formed high-quality crystals, while only the arginine mutant of Aim18-Nd70 formed crystals, presumably due to the increased stability (Fig. S3*B*).

We solved the crystal structures for Aim18p-Nd70-R123A (PDB: 8EW8) and Aim46p-Nd62-WT (PDB: 8EW9) to resolutions of 2.15 Å and 2.0 Å, respectively (Table S3). Both Aim18p-Nd70-R123A and Aim46p-Nd62-WT adopt the CHI fold, as determined by their RMSD values of 3.148 and 2.768 when superpositioned with CHI1 from *Medicago sativa* (MsCHI1) (Fig. 3, *A* and *B*; PDB: 1EYQ (2)). Almost the entirety of the core 7–stranded antiparallel β-sheet is shared between these proteins with small deviations in the more solvent-accessible α-helices (Fig. 3, *A* and *B*). Of note is a region of the Aim18p-Nd70 and Aim46-Nd62 structures that lies in between plant CHI α1 and α2. This region contains an extra loop that is absent in plant CHI domain–containing proteins (Fig. 3, *A* and *B* and S3*D*) but is conserved across multiple ascomycete Aim18p and Aim46p homologs (Fig. S3*E*).

Nearly all of the CHI catalytic residues are substituted nonconservatively in Aim18p and Aim46p except for the catalytic arginine (Fig. 3*C*), which is invariably conserved in fungal homologs of Aim18p and Aim46p (Fig. S3*C*). A single nonconservative mutation in these residues is sufficient to nearly abolish catalytic activity (5), so the presence of several substitutions suggests that Aim18p and Aim46p likely lack canonical CHI enzymatic activity. In contrast, Aim18p and Aim46p share 4/5 active site residues with the fatty acid–binding CHI-like protein, FAP1 from *Arabidopsis thaliana* (AtFAP1) (PDB: 4DOO (6); Figs. 3*C* and S3*C*).

**Figure S4:**
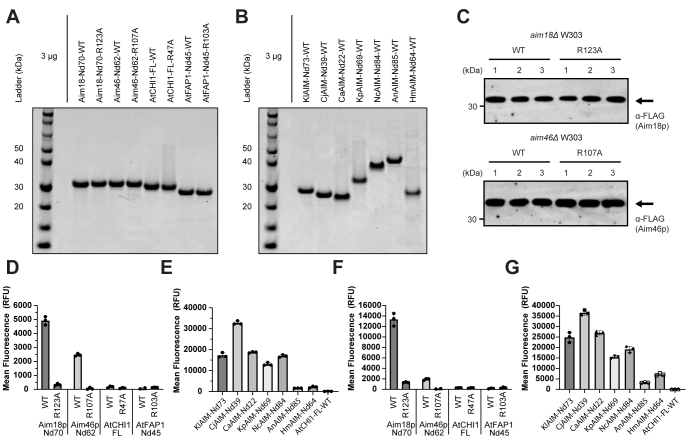


To test whether Aim18p and Aim46p possess either CHI or fatty acid–binding activities, we first designed protein expression constructs for the control proteins AtCHI1 and AtFAP1-Nd45 (full-length AtFAP1 did not express, much like the fungal CHI domain–containing proteins, which require an N-terminal truncation for purification). We performed the standard CHI assay on WT and catalytic arginine mutant constructs of Aim18p-Nd70, Aim46p-Nd62, and AtCHI1 (Figs. 3*D* and S4*A*). Only AtCHI-WT rapidly converted naringenin chalcone to naringenin, supporting the hypothesis that Aim18p and Aim46p lack CHI catalytic activity. We also performed differential scanning fluorimetry to assess changes in thermal stability on WT and catalytic arginine mutant constructs of Aim18p-Nd70, Aim46p-Nd62, AtCHI1, and AtFAP1-Nd45 as a proxy for lipid binding (6) (Figs. 3*E* and S4*A*). The positive control AtFAP1-Nd45-WT was the only protein stabilized by the addition of linolenic acid and palmitic acid, while the rest of the proteins were either not stabilized or were destabilized, a phenomenon also observed with AtCHI1 (6). If Aim18p and/or Aim46p possess fatty acid–binding activity similar to AtFAP1, we would expect these proteins to be stabilized by the addition of linolenic acid or palmitic acid; instead, we observe no change or a destabilizing interaction, suggesting that Aim18p and Aim46p do not have AtFAP1-like fatty acid–binding activity. Taken together, while Aim18p and Aim46p adopt the canonical CHI fold, the lack of CHI catalytic activity and the absence of fatty acid–binding activity suggest that fungal CHI domain–containing proteins likely have a different biological activity from plant CHI domain–containing proteins.

### Aim18p and Aim46p are hemoproteins

**Figure 4:**
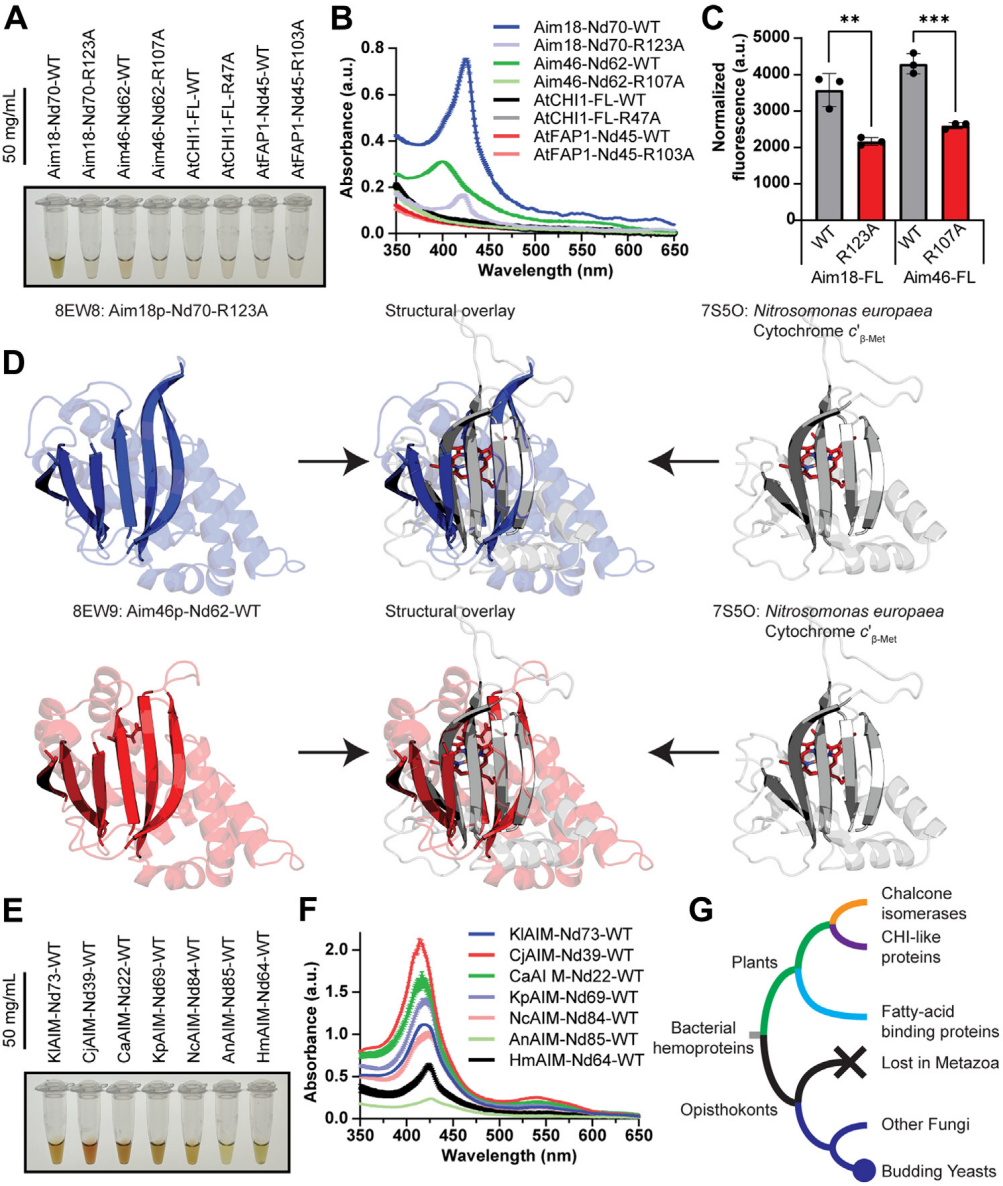
**Aim18p and Aim46p are hemoproteins.***A*, image of 50 mg/ml recombinant Aim18p-Nd70 and Aim46p-Nd62 variants copurifying with a golden color compared to AtCHI1 and AtFAP1. *B*, UV-Vis spectroscopy of proteins in (*A*) highlighting the presence of a Soret peak at ∼400 nm, a characteristic of hemoproteins. Mean ± SD, *n* = 3 independent technical replicate measurements of the same sample. *C*, fluorometric oxalic acid heme determination assay with immunoprecipitations of FLAG-tagged Aim18p and Aim46p expressed in *Saccharomyces cerevisiae* (∗∗*p* = 0.0062 Aim18-FL-WT vs Aim18-FL-R123A, ∗∗∗*p* = 0.0005 Aim46p WT vs Aim46p R107A; mean ± SD, *n* = 3 independent technical replicates). Average heme content measurements were normalized *via* densitometry by protein abundance (Fig. S4*C*). Significance was calculated by an unpaired, two-tailed Student’s *t* test. *D*, structural overlay of Aim18p (*blue*) and Aim46p (*red*) with cytochrome c'_β-Met_ from the bacterium *Nitrosomonas europaea* (*gray*, PDB: 7S5O). Alpha helices made slightly transparent to highlight homology of core antiparallel beta sheet for both aligned proteins. *E*, image of recombinant fungal AIM homologs copurifying with golden color. Recombinant fungal AIM homologs are named based on their species of origin (KlAIM = *Kluyveromyces lactis*; CjAIM = *Cyberlindnera jadinii*; CaAIM = *Candida albicans*; KpAIM = *Komagataella pastoris*; NcAIM = *Neurospora crassa*; AnAIM = *Aspergillus nidulans*; HmAIM = *Hypsizygus marmoreus*). *F*, UV-Vis spectroscopy of proteins in (*E*) highlighting the presence of a Soret peak at ∼400 nm, a characteristic of hemoproteins. Mean ± SD, *n* = 3 independent technical replicate measurements of the same sample. Recombinant fungal AIM homologs are named based on their species of origin (KlAIM = *K. lactis*; CjAIM = *C. jadinii*; CaAIM = *C. albicans*; KpAIM = *K. pastoris*; NcAIM = *N. crassa*; AnAIM = *A. nidulans*; HmAIM = *H. marmoreus*). *G*, proposed phylogenetic tree of CHI domain–containing proteins with bacterial hemoproteins as the outgroup. AIM, Altered Inheritance of Mitochondria; CHI, chalcone isomerase.

Our purified recombinant Aim18p-Nd70 and Aim46p-Nd62 from *E. coli* exhibited a golden color, suggesting a bound ligand (Figs. 4*A* and S4*A*). Interestingly, catalytic arginine mutants lacked this golden color. Spectroscopy revealed that the colored fungal CHI domain–containing proteins had a distinct peak around 420 nm (Figs. 4*B* and S4, *D*–*G*), which likely represents the signature Soret peak of hemoproteins (27).

We considered whether heme binding might be an artifact of expressing recombinant fungal CHI domain–containing proteins in *E. coli.* To assuage this concern, we generated centromeric yeast expression plasmids encoding FLAG-tagged, full-length Aim18p and Aim46p to test heme binding in their native organism, *S. cerevisiae*. Due to the lower yield of protein from these immunoprecipitations, we confirmed the presence of heme through an orthogonal approach. We used a fluorescent heme detection assay that uses heated oxalic acid to liberate the heme molecule of its central iron metal, generating a fluorescent protoporphyrin molecule. This experiment revealed that Aim18p and Aim46p coimmunoprecipitated with bound heme and that mutating the catalytic arginine significantly reduced heme binding (Figs. 4*C* and S4*C*). Despite multiple attempts, we were unable to generate heme-bound crystal structures for either Aim18p or Aim46p. Additionally, the structure of Aim46p-Nd62-WT contained a small molecule resembling α-ketoglutarate in the active site (Document S2), but we were unable to confirm its identity *via* mass spectrometry.

The tertiary structure of the CHI domain is classified as a mixed α+β fold. To explore whether other hemoproteins possess an α+β fold similar to Aim18p and Aim46p, we searched our structures against the Dali server (28). A heme-c–binding protein, cytochrome *c*'_β-Met_ from the ammonia-oxidizing bacterium *Nitrosomonas europaea* (PDB: 7S5O; (29)), was among the top hits outside of the plant CHI domain–containing proteins. Alignments of the Aim18-Nd70-R123A and Aim46-Nd62-WT structures with the bacterial protein revealed that, while the α-helices differed substantially, the core CHI antiparallel β-sheet was structurally homologous to the core antiparallel β-sheet of cytochrome *c*'_β-Met_ (Fig. 4*D*).

The presence of a bacterial hemoprotein with structural similarities to the CHI fold suggests the possibility that the ancestral CHI protein was a hemoprotein. To further determine the phylogenetic breadth of heme binding, we leveraged the rich dataset of fungal CHI domain–containing proteins (Fig. S1*A*) to find extant fungal CHI domain–containing proteins representing several clades of fungi (Fig. S3*C*), including at least one representative from the phylum Basidiomycota and all three subphyla of the phylum Ascomycota. With these sequences, we designed N-terminally truncated (18) protein expression constructs for these fungal CHI domain–containing proteins. We observed that all successfully purified recombinant fungal CHI domain–containing proteins copurified with heme (Figs. 4, *E* and *F* and S4, *B*, *D*–*G*). Collectively, these data suggest that the ancestral fungal CHI domain–containing protein was a hemoprotein and that the entire CHI domain–containing protein family might share a common ancestor with a family of bacterial hemoproteins (Fig. 4*G*).

## Discussion

CHI domain–containing proteins (CHIs) comprise a large family of proteins that feature a common fold but wield multiple functions. This family derives its name from enzymes that catalyze the intermolecular cyclization of chalcones to flavanones (Type I and Type II CHIs; (2, 30)), but this family also includes fatty acid–binding proteins (Type III CHIs; (6)) and noncatalytic proteins that are thought to increase flavonoid biosynthesis (Type IV CHIs; (31, 32)). In this study, we expand the functional repertoire of the CHI domain–containing protein family by revealing that the fungal clade of CHIs are hemoproteins that lack canonical CHI activity. Moreover, given that gene duplications are less widespread among budding yeasts than among the CHI domain–containing proteins seen in plants, it is likely that these fungal sequences are more conserved in function and may be more functionally similar to the ancestral protein (Fig. S1*A*).

Despite these advances, without an *in vivo* phenotype or heme-bound crystal structure, it is difficult to ascertain how Aim18p and Aim46p employ their CHI fold and heme-binding capabilities in mitochondria. Hemoproteins have multiple functions, including, but not limited to, ligand delivery (*e.g.*, oxygen *via* hemoglobin), signal transduction (*e.g.*, nitric oxide binding by soluble guanylyl cyclases), electron transport, and catalysis (*e.g.*, peroxidases and monooxygenases) (33, 34).

The CHI fold is classified as an α+β fold, which comprises a mostly antiparallel beta-sheet decorated with alpha helices (35). α+β hemoproteins (including Aim18p and Aim46p) comprise approximately 10% of all known hemoprotein folds (36, 37) and often leverage their heme for environmental sensing and heme transport functions. These include the Per-Arnt-Sim domain–containing hemoproteins (38, 39), such as the oxygen sensors Dos and FixL (40, 41). Other examples of α+β hemoproteins are the heme acquisition protein HasA in bacteria (42, 43) and the SOUL family of hemoproteins, which have proposed biological functions ranging from regulating cell fate to heme transport (44). There are exceptions to this theme, including the multifunctional α+β ferredoxin proteins (45, 46), but the majority of α+β hemoproteins are gas sensors or heme transporters. While we do not know the exact function of Aim18p and Aim46p, it is reasonable to speculate that these proteins might regulate mitochondrial function by utilizing heme in a similar manner.

We observed that the heme binding of recombinant Aim46p, when purified from *E. coli*, was more variable across preps (Fig. S4, *D* and *F*). This suggests that heme binding might be sensitive to environmental fluctuations and perhaps that Aim18p and/or Aim46p might serve as heme-sensing proteins, although this remains speculation. Although our data show that the conserved CHI arginine does influence heme binding (Figs. 4, *A*–*C* and S4, *D* and *F*) in Aim18p and Aim46p, it is most likely not the only heme-coordinating residue; future experiments will be necessary to fully understand the coordination environment between heme and the CHI domain.

Our investigation of Aim18p and Aim46p is part of the grand post-genomic era effort to define functions for all proteins (47, 48). Modern structural biology approaches and the advent of powerful structural prediction algorithms doubtlessly are advancing us toward this goal (49, 50, 51). However, our work serves as a reminder that biochemical functions are often not immediately evident from structure. We demonstrate that the CHI domain–containing hemoproteins in fungi do not behave like their plant relatives, even though they have high sequence and structural homology. Protein folds conferring multiple functions is a well-established biological phenomenon (52); for example, thousands of proteins contain a Rossman-like fold, but their substrates, cofactors, and functions can differ significantly (53). Other examples of many include the α+β barrel hemoproteins, which share a ferredoxin-like fold, but have diverse functions (45, 46), as well as cytochrome P460 (an enzyme) and cytochrome c′_β_ (a gas-binding protein), which adopt a similar fold but bind different substrates (54).

To our knowledge, this is the first published biochemical study of a CHI domain–containing protein in the fungal kingdom. Our analyses assign a distinct heme-binding function to the previously uncharacterized proteins Aim18p and Aim46p in *S. cerevisiae*, as well as to a diverse array of fungal homologs, expanding previous observations that the CHI domain is functionally plastic (6, 7, 31). We also suggest, through structural homology prediction, that the CHI domain might be related to bacterial cytochrome P460s and suggest that the ancestral CHI-like proteins may have relied on heme-binding for its function. Collectively, our study provides a foundation for further work to define the evolution and specific *in vivo* roles of Aim18p and Aim46p.

## Experimental procedures

### Evolutionary analysis

Flavonoid biosynthesis proteins from *A. thaliana* were used to search for homologs in *S. cerevisiae* using DELTA-BLAST with an e-value < 1e-15. To search for CHI homologs in yeasts, a HMMER profile was constructed using *S. cerevisiae* Aim18p and Aim46p and used to conduct a HMMER search 332 annotated budding yeast genomes (15) to identify hits with score >50 and e-value <0.001. To search for CHI homologs in more divergent fungi, tBLASTn was used to identify hits in over 700 unannotated Ascomycota genomes (16) with e-value <1e-6. The full plant and fungal CHI homolog phylogeny was built by adding the fungal sequences to a structural alignment of plant CHI homolog sequences (7) using the —add function of MAFFT (55) using default settings. The resulting alignment was truncated to the regions matching the initial structural alignment that included the CHI domain and filtered to sites with less than 50% gaps using trimAl (56). A phylogenetic tree was constructed using fasttree (57) with the settings (d -lg -gamma -spr 4 -slownni -mlacc 2), midpoint rooted, and edited to collapse clades using iTOL (58). The absence of any CHI homologs in metazoan lineages was determined by repeated search attempts using both blastp and DELTA-BLAST at permissive e-value thresholds restricted to relevant taxonomic groups. The determination that the single CHI homolog in the published genome of *S. arboricola* was due to a fusion of paralogs was made by aligning the coding sequence of all hits from *Saccharomyces* species *via* MAFFT using default settings (55) and looking for signatures of recombination between *AIM18* and *AIM46* homologs in the *S. arboricola* gene using RDP4 (59). A single recombination event was found in this sequence (all tests of recombination were significant at p<1e-13), which supports a fusion with *AIM46* providing roughly the 5′ 60% and *AIM18* the final 3′ 40% of the sequence.

A subset of protein sequences of plant and fungal CHIs were aligned *via* MAFFT using default settings (55). Protein sequence alignments were visualized using Jalview 2.11.2.5 and colored by sequence conservation (60). Mid-point–rooted phylogenetic trees of plant and fungal CHIs were generated through the Phylogeny.fr “one click” mode workflow (http://phylogeny.lirmm.fr/) using default settings (61, 62, 63, 64, 65). Alignments and phylogenetic trees were exported as svg files and annotated in Adobe Illustrator 26.5.

### Yeast strains and cultures

All yeast strains used in this study were (or were derived from) WT *Saccharomyces cerevisiae* haploid W303-1A (*MAT*a, *his3 leu2 met15 trp1 ura3*). Yeast single and double deletions of *aim18* and/or *aim46* were generated by replacing the ORFs with the *HIS3MX6* cassette *via* homologous recombination, as previously described (66) (Table S2). Homologous recombination donor DNA was ∼5 μg *HIS3MX6* cassette amplified with 40 bp homology upstream and downstream of the corresponding ORF. Successful insertion of the KO cassette was confirmed by a PCR-based genotyping assay.

Unless otherwise described, yeast strains were cultured in synthetic complete (SC) (or synthetic complete minus uracil (Ura^-^), for plasmid-based experiments) medium with the indicated carbon source. SC medium contained drop-out mix complete (without yeast nitrogen base, US Biologicals: D9515), yeast nitrogen base (without amino acids, carbohydrate, or ammonium sulfate, US Biologicals: Y2030), 5 g/l ammonium sulfate, and the indicated carbon source (w/v). SC Ura^-^ medium contained drop-out mix synthetic minus uracil (without yeast nitrogen base, US Biologicals: D9535), yeast nitrogen base (without amino acids, carbohydrate, or ammonium sulfate, US Biologicals: Y2030), 5 g/l ammonium sulfate, and the indicated carbon source (w/v). All media pH values were adjusted to 6.0 and sterilized *via* vacuum filtration (0.22 μM).

### Plasmid cloning

Yeast expression plasmids were cloned *via* standard restriction enzyme and ligation methods. ORF-specific inserts for *AIM18* and *AIM46* (and their respective arginine mutants) with 20 bp homology to p416-GPD (Elizabeth Craig Lab) were amplified from W303 yeast genomic using primers specific to each ORF (Table S2). PCR-amplified ORF inserts were ligated into *Xba*I- and *Sal*I-HF–digested p416 GPD and then then transformed into *E. coli* 10 G chemically competent cells (Lucigen). Successful transformants were miniprepped and confirmed *via* Sanger sequencing (UW–Madison Biotechnology DNA sequencing core facility). For experiments, yeast strains were transformed with ∼300 ng plasmid *via* the LiAc/SS-DNA/PEG method (67).

*E. coli* protein expression plasmids were cloned *via* standard Gibson assembly. ORF inserts with 40 bp homology to pET-21a(+) were amplified from W303 yeast genomic DNA (for *AIM18* and *AIM46*) or gBlocks (for *AtCHI1*, *AtFAP1*, and fungal *AIM* homologs) using 60 bp primers specific to each ORF (Table S2). gBlocks for fungal *AIM* homologs included *AIM* ORFs from *Kluyveromyces lactis*, *Cyberlindnera jadinii, Candida albicans, Komagataella pastoris, Yarrowia lipolytica, Lipomyces starkeyi*, *Neurospora crassa*, *Aspergillus niger, Schizosaccharomyces pombe,* and *Hypsizygus marmoreus* (Table S2; Document S1). PCR-amplified ORF inserts were Gibson assembled into *Nco*I-HF– and *Xho*I-digested pET-21a(+) (Addgene) and then transformed into *E. coli* 10 G chemically competent cells (Lucigen). Successful transformants were miniprepped and confirmed *via* Sanger sequencing (UW–Madison Biotechnology DNA sequencing core facility).

### Site-directed mutagenesis

Site-directed mutagenesis reactions to generate catalytic arginine point mutants for *AIM18* and *AIM46* were performed according to the Q5 Site-Directed Mutagenesis Kit (New England Biolabs, Table S2). Site-directed mutagenesis reactions were transformed into *E. coli* 10 G chemically competent cells (Lucigen). Successful transformants were miniprepped and confirmed *via* Sanger sequencing (UW–Madison Biotechnology DNA Sequencing Core Facility). Arginine point mutants for *AtCHI1* and *AtFAP1* were ordered as separate gBlocks.

### Yeast drop assay

Single colonies of *S. cerevisiae* W303-1A were used to inoculate 3-ml starter cultures of SC +2% glucose (w/v) medium, which were incubated overnight spinning on culture tube wheel (20 h, 30 °C, max speed). Yeast from these starter cultures were serially diluted (10^5^, 10^4^, 10^3^, 10^2^, and 10^1^) in water. Four microliters of each serial dilution were dropped onto solid agar SC plates containing either 2% glucose (w/v), 3% glycerol (w/v), or 2% EtOH (v/v) and incubated (2–4 days, 30 °C).

### Yeast petite frequency assay

*S. cerevisiae* W303-1A were streaked onto nonfermentable YPEG plates to eliminate petites (1% yeast extract [w/v], 2% peptone [w/v], 3% EtOH [w/v], 3% glycerol [w/v], and 2% agar [w/v]). Single colonies of petite-free *S. cerevisiae* W303-1A were used to inoculate 3-ml fermentation starter cultures of SC +2% glucose (w/v) medium, which were incubated overnight spinning on a culture tube wheel to remove selection against petite formation (30 °C, maximum speed). Yeast cells from these starter cultures were then plated for single colonies on YPDG plates (1% yeast extract [w/v], 2% peptone [w/v], 0.1% glucose [w/v], 3% glycerol [w/v], and 2% agar [w/v]) (2–3 days, 30 °C). Petite colonies were identified and counted manually, and the resulting petite frequencies were recorded in Microsoft Excel and analyzed in Prism 9.2.0 (GraphPad by Dotmatics).

### Crude mitochondria purification

Single colonies of *S. cerevisiae* W303-1A were used to inoculate 3-ml starter cultures of either SC +2% glucose (w/v) or Ura^-^ +2% glucose (w/v) (for plasmid-transformed strains) medium, which were incubated overnight spinning on a culture tube wheel (20–24 h, 30 °C, max speed). Starter cultures of yeast strains were used to inoculate 500-ml respiratory cultures of either SC +3% glycerol (w/v) +0.1% glucose (w/v) or Ura^-^ +3% glycerol (w/v) +0.1% glucose (w/v) (for plasmid-transformed strains) medium with 2.5 × 10^7^ cells, which were incubated until past the diauxic shift (25 h, 30 °C, 225 rpm). For whole-cell analysis, 1 × 10^8^ cells were collected, harvested by centrifugation (21,130*g*, 3 min, room temperature [RT]), flash frozen in N_2_(l), and stored at −80 °C. The remaining culture was harvested by centrifugation (4000*g*, 10 min, RT), washed with 25 ml ddH_2_O, and then weighed (∼2 g). Yeast crude mitochondria were then isolated as previously described (68). Isolated yeast crude mitochondria were gently resuspended (using a wide-bore pipette tip) in SEM buffer (250 mM sucrose, 1 mM EDTA, 10 mM MOPS-KOH, pH 7.2, 4 °C). Total protein concentration for whole-cell and isolated yeast crude mitochondria samples were quantified for total protein using the Pierce BCA Protein Assay Kit (Thermo Fisher Scientific). Isolated yeast crude mitochondria were aliquoted as 40 μg aliquots, flash frozen in N_2_(l), and stored at −80 °C.

### Yeast FLAG immunoprecipitation

Single colonies of *S. cerevisiae* W303-1A expressing full-length Aim18p or Aim46p under control of the glyceraldehyde-3-phosphate dehydrogenase gene (THD3) promoter were used to inoculate 3-ml starter cultures of Ura^-^ +2% glucose (w/v) medium, which were incubated overnight spinning on a culture tube wheel (24 h, 30 °C, maximum speed). Starter cultures of yeast strains were used to inoculate 100-ml respiratory cultures of Ura^-^ +3% glycerol (w/v) +0.1% glucose (w/v) (for plasmid-transformed strains) medium supplemented with 500 μM ammonium iron (II) sulfate hexahydrate ([Argos Organics: 201370250], as iron is limiting in synthetic media) with 5 × 10^6^ cells, which were incubated until past the diauxic shift (34 h, 30 °C, 225 rpm). Yeast cultures were harvested by centrifugation (4000*g*, 10 min, RT), washed with 25 ml ddH_2_O, transferred to a 2 ml screw-cap Eppendorf tube, and pinned *via* centrifugation (21,130*g*, 2 min, RT). Yeast pellets were resuspended in 400 μl FLAG lysis buffer (20 mM Hepes, pH 7.4, 100 mM NaCl, 10% [w/v] glycerol, 3% [w/v] digitonin [Sigma: D141], 1 mM DTT, 1× PI-1 [10 μM benzamidine, 1 μg/ml 1,10-phenanthroline], 1× PI-2 [0.5 μg/ml each of pepstatin A, chymostatin, antipain, leupeptin, aprotinin]), and 200 μl glass beads. Yeast pellet resuspensions were lysed *via* bead beating on a Disruptor Genie (Zymo Research) cell disruption device (3×, 2 min, with 2 min rests in between bead beatings). Yeast lysates were clarified *via* centrifugation (21,130*g*, 15 min, 4 °C) and transferred to a new tube. Equal volumes of clarified yeast lysates (400 μl) were incubated with 20 μl Anti-FLAG M2 Magnetic Beads (Millipore: M8823). Magnetic beads containing FLAG-tagged protein were washed four times with FLAG wash buffer (20 mM Hepes, pH 7.4, 100 mM NaCl, 0.05% [w/v] digitonin [Sigma: D141], and 10% [w/v] glycerol) and eluted with FLAG elution buffer (20 mM Hepes, pH 7.4, 100 mM NaCl, 0.05% [w/v] digitonin [Sigma: D141], 10% [w/v] glycerol, and 0.4 mg/ml, and 1× FLAG-peptide [Sigma: F3290]) (30 min, with moderate agitation, RT). 0.5% of each elution was analyzed *via* western blot as described below to check for equal protein expression and immunoprecipitation. For heme measurement assays, protein abundances were normalized to the first replicate of WT W303-1A yeast *via* densitometry with the ImageJ 1.53c software ((69), NIH).

### Proteinase K protection assay

Proteinase K protection assays were performed as previously described (70, 71). Forty micrograms yeast crude mitochondria were incubated with digitonin (0%, 0.01%, 0.02%, 0.04%, 0.06%, 0.08%, and 0.1% [w/v]) and 1% Triton (w/v) in the presence of 300 μg/ml proteinase K (40 μl reaction size, 15 min, RT). Proteinase K was then inhibited with the addition of 7 mM PMSF. Each reaction was precipitated with 25% trichloroacetic acid (20 min, on ice) and resuspended in in 40 μl 1× lithium dodecyl sulfate (LDS) sample buffer containing 4% β-mercaptoethanol (BME). Ten microliters of each reaction was analyzed *via* western blot as described below.

### Sodium carbonate extraction assay

Sodium carbonate extraction assays were performed as previously described (24). Forty micrograms yeast crude mitochondria were incubated with 100 μl of 100 mM sodium carbonate (Thermo Fisher Scientific: S263, 30 min, on ice). Soluble and insoluble fractions were isolated *via* centrifugation (90,000*g*, 30 min, 4 °C) in an Optima MAX-OP (Beckman Coulter) centrifuge using the TLA-100.3 rotor. Pellets (insoluble fraction) and supernatants (soluble fraction) were precipitated with 25% trichloroacetic acid (20 min, on ice) and resuspended in in 40 μl 1× LDS sample buffer containing 4% BME. Ten microliters of each reaction was analyzed *via* western blot as described below.

### Western blotting

Protein samples in 1× LDS sample buffer containing 4% BME were separated by size on 4 to 12% Novex NuPage Bis-Tris SPS-PAGE (Invitrogen) gels (2 h, 100 V). Gels were transferred to a Immobilon-FL PVDF Membranes (Millipore) using the Mini Blot Module western blotting system (Invitrogen) for 1 h in transfer buffer (192 mM glycine, 25 mM Tris, 20% methanol [v/v], 4 °C). Membranes were dried for 15 min in the fume hood, rehydrated with a 15 s methanol wash, and submerged in TBST (500 mM NaCl, 20 mM Tris pH 7.4, 0.05% Tween 20 [v/v]). Membranes were blocked in 5% nonfat dry milk (NFDM) in TBST for 1 h on a rocker at RT. Membranes were incubated with primary antibodies diluted (Table S2) in 1% NFDM in TBST with 0.02% (w/v) sodium azide overnight on a rocker at 4 °C. After the primary incubation, membranes were washed in TBST (3×, 5 min, RT) on a rocker. Membranes were incubated on a rocker with fluorophore-conjugated secondary antibodies diluted (Table S2) in 5% NFDM in TBST (1 h, RT). After the secondary incubation, membranes were washed in TBST (3×, 5 min, RT) on a rocker and imaged on LI-COR Odyssey CLx using the Image Studio v5.2 software (LI-COR). Special care was made to ensure that membranes were exposed to the light as little as possible to protect the fluorescent signal of the secondary antibody.

### Antibodies

Proteins of interest were detected with primary antibodies to Tom70p (72) (a gift from Nora Vögtle, 1:500), GAPDH (Thermo Fisher Scientific, MA5-15738, 1:4000), FLAG (Proteintech, 66008-3-lg, 1:5000), Cytb2 (73) (a gift from Elizabeth Craig, 1:1000), Tim23 (74) (a gift from Elizabeth Craig, 1:5000), Tim44 (75) (a gift from Elizabeth Craig, 1:1000), and Cit1 (76) (Biomatik, 1:4000). Fluorescent-conjugated secondary antibodies included α-mouse 800 (LICOR, 926-32210, 1:15000) and α-rabbit 800 (LICOR, 926-32211, 1:15000). Detailed information about antibodies is in Table S2.

### Purification of recombinant proteins

pET-21a(+) constructs containing DNA-encoding N-terminal 8X-His-tagged CHI domain–containing proteins were transformed into BL21-CodonPlus (DE3)-RIPL Competent Cells (Agilent) for recombinant protein expression. Proteins were gently expressed overnight by autoinduction (77) (37 °C, 3 h; 20 °C, 20 h, 225 rpm). Cells were harvested *via* centrifugation (4000*g*; 30 min, RT), flash frozen in N_2_(l), and stored at −80 °C. For purification, pellets expressing recombinant protein were thawed on ice for at least an hour with lysis buffer (400 mM NaCl, 160 mM Hepes (pH 7.5), 5 mM BME, 0.25 mM PMSF, and 1 mg/ml lysozyme). Thawed pellets were resuspended *via* vortexing until the solution was homogenous. Resuspended pellets were lysed *via* sonication (3×, 70% amplitude, 20 s, with 60 s rests) and clarified *via* centrifugation (15000*g*, 30 min, 4 °C). Clarified lysates were incubated with TALON metal affinity resin (Takara Bio USA) for 1 h with end-over-end rotation at 4 °C. Resin was pelleted *via* centrifugation (700*g*, 2 min, 4 °C), washed four times with equilibration buffer (400 mM NaCl, 160 mM Hepes (pH 7.5), 5 mM BME, 0.25 mM PMSF), and washed twice with wash buffer (400 mM NaCl, 160 mM Hepes (pH 7.5), 5 mM BME, 0.25 mM PMSF, 40 mM imidazole). Proteins were eluted from the resin with elution buffer (400 mM NaCl, 160 mM Hepes (pH 7.5), 5 mM BME, 0.25 mM PMSF, 400 mM imidazole). Eluted proteins were buffer-exchanged twice into equilibration buffer (400 mM NaCl, 160 mM Hepes (pH 7.5), 5 mM BME, 0.25 mM PMSF) and concentrated to ∼500 μl with an Amicon Ultra-15 Centrifugal Filter Unit (10 kDa cutoff, Millipore). Concentrated eluted proteins were cleared of protein precipitate *via* centrifugation (21,130*g*, 15 min, 4 °C). Protein concentrations were determined *via* Bradford assay (Bio-Rad Protein Assay Kit II; (78)), diluted with equilibration buffer (400 mM NaCl, 160 mM Hepes (pH 7.5), 5 mM BME, 0.25 mM PMSF) to the desired concentration, aliquoted into strip tubes, flash frozen in N_2_(l), and stored at −80 °C.

### Size-exclusion chromatography

For crystallography studies, concentrated protein elutions (before the protein concentration quantification step above) were passed through a 0.22 μM filter and injected into the loading loop of a NGC Medium-Pressure Chromatography System (Bio-Rad) fitted with a HiLoad 16/600 Superdex 75 pg column. Proteins were separated *via* SEC using SEC buffer (50 mM NaCl, 20 mM Hepes (pH 7.5), 0.3 mM TCEP) in 1 ml fractions. Fractions from SEC were analyzed *via* SDS-PAGE, and the major fractions containing the target protein were pooled and then concentrated with an Amicon Ultra-15 Centrifugal Filter Unit (10 kDa cutoff, Millipore) to ∼500 ml. Concentrated SEC fractions were then dialyzed against crystal buffer (5 mM Hepes, 400 mM NaCl, 0.3 mM TCEP, pH 8.0) in a Slide-A-Lyzer MINI Dialysis Device (3.5 kDa cutoff, Thermo Fisher Scientific) overnight on an orbital shaker at 4 °C. Protein concentrations were determined *via* Bradford assay (Bio-Rad Protein Assay Kit II; Bradford 1976), diluted with crystal buffer (5 mM Hepes, 400 mM NaCl, 0.3 mM TCEP, pH 8.0) to 20 mg/ml, aliquoted into strip tubes, flash frozen in N_2_(l), and stored at −80 °C.

### Crystallization, X-ray diffraction, and refinement

Crystallization experiments and structure determination were conducted in the Collaborative Crystallography Core in the Department of Biochemistry at the University of Wisconsin. All crystallization screens and optimizations were conducted at 293K in MRC SD-2 crystallization plates, set with a STP Labtech Mosquito crystallization robot. Hampton IndexHT and Molecular Dimensions JCSG+ were used as general screens. Crystals were cooled by direct immersion in liquid nitrogen after cryopreservation and harvest using MiTeGen MicroMounts. X-ray experiments were conducted at the Advanced Photon Source, Argonne National Lab, GMCA@APS beamline 23ID-D. Diffraction data were collected on a Pilatus 3-6M detector and reduced using XDS (79) and XSCALE (80). Structure solution and refinement used the Phenix suite of crystallography programs (81). Iterative rounds of map fitting in Coot (82) and phenix.refine (83) were used to improve the atomic models. MOLPROBITY (84) was used to validate the structures. Data collection and refinement statistics for the structures can be found in Table S3.

The best crystals of SeMet Aim18p-Nd70-R123A grew from 26% PEG 3350, 0.2 M Li_2_SO_4_, 0.1 M Na-Hepes buffer, pH 7.5. Crystals were cryopreserved by supplementing the PEG concentration to 30%. Diffraction data extending to 2.2 Å was collected on 2018-02-07 at energies near the SeK-edge (peak = 0.97937 Å, edge = 0.97961 Å, and high remote at 0.96437 Å). Phenix.hyss (85) located the expected 5 Se sites from one copy of the protein per asymmetric unit in space group P3_2_21. The structure was phased and traced using Phenix.autosol (86) with a phasing figure of merit = 0.47 and map skew = 0.6. The initial chain trace was continuous from residue 88 to the C-terminus. The final model starts at residue 83 and includes five sulfate ions per chain.

The best crystals of Aim46p-FL-WT were grown using microseeds obtained from a similar condition. The seeds were stabilized in 30% PEG 2000, 0.1 M MES pH 6.5. The droplet was composed of 20 nl microseed suspension, 180 nl Aim46p-Nd62-WT, and 300 nl PEG 2000, 0.1 M MES, pH 6.5. Crystals were cryoprotected with 30% PEG 2000. Data were collected on 2018-12-08 at 1.0332 Å. Data extended to 2.0 Å and belonged to space group P2_1_. One copy of the protein per asymmetric unit was by molecular replacement using Phaser starting from an appropriately pruned Aim18p-Nd70-R123A model (log-likelihood gain 307, translation function z-score 11.7). The final model is continuous from residue 70 to the C-terminus and includes a ligand modeled as α-ketoglutarate.

### CHI assay

The CHI activity assay was performed as previously described (4, 5, 7). CHI activity assays were run with the following reaction mixture: 150 mM NaCl, 50 mM Hepes (pH 7.5), 5% EtOH (as a cosolvent), and 200 μM naringenin chalcone (Aldlab Chemicals: AT15178). To initiate the reaction, 10 nM protein was added, mixed, and immediately run on an Infinite M1000 multimode microplate reader (Tecan). Conversion of naringenin chalcone (yellow) into naringenin (colorless) was monitored by the depletion of absorbance at 381 nm. Absorbance measurements at 381 nm were taken every 10 s for 30 m. The resulting absorbance data were exported to Microsoft Excel and analyzed in Prism 9.2.0 (GraphPad by Dotmatics). A no protein (buffer and substrate only) control is necessary for these experiments because naringenin chalcone nonenzymatically cyclizes into naringenin at a slow rate over the course of the assay.

### Differential scanning fluorimetry

The general differential scanning fluorimetry method has been documented previously (87). For screening fatty acid binding, 20 μl reactions containing 5 μM protein, 50 μM fatty acid from 10 mM stock in DMSO or equivalent vehicle (Linolenic acid [Sigma: L2376]) and Oleic acid [Sigma: O1008]), 100 mM Hepes pH 7.5, 150 mM NaCl, and 10× Sypro Orange dye (Thermo Fisher Scientific: S6651) were made in MicroAmp Optical 96-well reaction plates (Thermo Fisher Sceintific: N8010560), centrifuged (200*g*, RT, 30 s), and incubated at RT in the dark for 1 h. Fluorescence was then monitored with the ROX filter using a QuantStudio 3 Real-Time PCR system along a temperature gradient from 4 to 95 °C at a rate of 0.025 °C/s. Protein Thermal Shift software v1.4 (Applied Biosystems; https://www.thermofisher.com/us/en/home/technical-resources/software-downloads/protein-thermal-shift-software.html) was used to determine T_m_ values by determining the maximum of first derivative curves. Melt curves flagged by the software were manually inspected. Each fatty acid-protein pair was run in triplicate, and error bars represent SDs. ΔT_m_ values were determined by subtracting the average of matched vehicle controls from the same plate.

### UV-Vis spectroscopic measurement

Purified proteins were assessed for the presence or absence of the Soret peak (27). 1.5 μl of 50-mg/ml purified recombinant protein were analyzed on a NanoDrop 2000c spectrophotometer (Thermo Fisher Scientific). Absorbance spectra from 350 nm to 700 nm (1 nm steps) were collected. The resulting absorbance spectra data were analyzed in Prism 9.2.0 (GraphPad by Dotmatics).

### Fluorometric heme measurement

A modified version of the fluorometry heme measurement assay was performed as described (88, 89). Hundred microliters of 2 M oxalic acid were added to 100-μL samples of 16 μM-purified protein or 100-μL FLAG immunoprecipitation elutions from *S. cerevisiae*. The resulting 200-μL reaction was split into two replicate 100-μL tubes (samples arrayed in a strip tube). The first replicate tube was kept in the dark at RT, and the other replicate tube was incubated at 98 °C for 30 min in a Mastercycler X50s thermocycler (Eppendorf). Both replicate samples were transferred to a black-walled microplate, and fluorescence measurements (λ_ex_ = 400 nm; λ_em_ = 620 nm) were taken on an Infinite M1000 multimode microplate reader (Tecan). The resulting fluorescence data were exported to Microsoft Excel and analyzed in Prism 9.2.0 (GraphPad by Dotmatics). Unheated samples were subtracted from the heated samples to produce the final fluorescence values and reported in relative fluorescence units.

### Lipid extraction from yeast

Starter cultures (3 ml YPD) were inoculated with an individual colony of yeast and incubated (30 °C, 230 rpm, 12 h). YPGD medium (100 ml medium at ambient temperature in a sterile 250-mL Erlenmeyer flask) was inoculated with 2.5 × 10^6^ yeast cells and incubated (30 °C, 230 rpm, 18 h). Samples were harvested 18 h after inoculation, a time point that corresponds to early respiration growth. 1 × 10^8^ yeast cells were harvested by centrifugation (3000*g*, 3 min, 4 °C) in screw-cap tubes, the supernatant was removed, and the cell pellet was flash frozen in N_2_(l) and stored at −80 °C. 100 μl of glass beads and 50 μl of 150 mM KCl and 600 μl of methanol with 0.1 μM CoQ_8_ internal standard (Avanti Polar Lipids) were added, and the cells were lysed *via* bead beating on a Disruptor Genie (Zymo Research) cell disruption device (2×, 5 min). To extract lipids, 400 μl of petroleum ether was added to samples and subjected to bead-beating for 3 min. Samples were then centrifuged (1000*g*, 2 min, 4 °C), and the ether layer (top) was transferred to a new tube. Extraction was repeated; the ether layers were combined and dried under argon gas at room temperature.

### Lipid Measurement by LC-MS

Extracted dried lipids were resuspended in 50 μl of mobile phase (78% methanol, 20% isopropanol, 2% 1 M ammonium acetate pH 4.4 in water) and transferred to amber glass vials with inserts. LC-MS analysis was performed using a Thermo Vanquish Horizon UHPLC system coupled to a Thermo Exploris 240 Orbitrap mass spectrometer. For LC separation, a Vanquish binary pump system (Thermo Fisher Scientific) was used with a Waters Acquity CSH C18 column (100 mm × 2.1 mm, 1.7 mm particle size) held at 35 °C under 300 μl/min flow rate. Mobile phase A consisted of 5 mM ammonium acetate in ACN:H_2_O (70:30, v/v) containing 125 μl/l acetic acid. Mobile phase B consisted of 5 mM ammonium acetate in IPA:ACN (90:10, v/v) with the same additive. For each sample run, mobile phase B was initially held at 2% for 2 min and then increased to 30% over 3 min. Mobile phase B was further increased to 50% over 1 min and 85% over 14 min and then raised to 99% over 1 min and held for 4 min. The column was re-equilibrated for 5 min at 2% B before the next injection. Five microliters of the sample were injected by a Vanquish Split Sampler HT autosampler (Thermo Fisher Scientific), while the autosampler temperature was kept at 4 °C. The samples were ionized by a heated ESI source kept at a vaporizer temperature of 350 °C. Sheath gas was set to 50 units, auxiliary gas to 8 units, sweep gas to 1 unit, and the spray voltage was set to 3500 V for positive mode and 2500 V for negative mode. The inlet ion transfer tube temperature was kept at 325 °C with 70% RF lens. For targeted analysis, the MS was operated in positive parallel reaction monitoring mode with polarity switching acquiring scheduled, targeted scans to CoQ intermediates: PPHB, DMQ, and CoQ. MS acquisition parameters include resolution of 15,000, stepped HCD collision energy (25%, 30% for positive mode and 20%, 40%, 60% for negative mode), and 3 s dynamic exclusion. Automatic gain control targets were set to standard mode.

### Data analysis

The resulting CoQ intermediate (oxidized form) data were processed using TraceFinder 5.1 (Thermo Fisher Scientific).

### Dali server protein structure comparison

The crystal structures of Aim18p-Nd70-R123A and Aim46p-Nd62-WT were submitted as query protein structures to the Dali server (http://ekhidna2.biocenter.helsinki.fi/dali/; (28)) and compared against all of the structures in the Protein Data Bank (PDB). PDB protein structural matches were provided in a list sorted by Z-score.

### PyMOL protein structure visualization

Protein crystal structures (pdb format) were imported into the PyMOL 2.4.0 software (https://pymol.org/2/). Proteins were structurally aligned using the super command and colored for visual clarity. In some cases, regions of proteins were made slightly transparent to highlight areas of importance. Scenes were exported using the ray command (specifically, ray_trace_mode,3) as png files and annotated in Adobe Illustrator 26.5.

### Statistical analysis

Statistical analyses were calculated with Microsoft Excel and visualized with Prism 9.2.0 (GraphPad by Dotmatics). For all experiments, “mean” indicates the arithmetic mean, SD indicates the standard deviation, and “*n”* indicates independent biological or technical replicates, as specified. *p*-values were calculated using a two-tailed, unpaired Student’s *t* test (for significance reporting: n.s. = *p* > 0.05; ∗ = *p* ≤ 0.05; ∗∗ = *p* ≤ 0.01; ∗∗∗ = *p* ≤ 0.001).

## Data availability

All data are contained within the article or supporting information. The structures presented in this article have been deposited in the Protein Data Bank (PDB) with the codes 8EW8 (Aim18p-Nd70-R123A) and 8EW9 (Aim46p-Nd62-WT).

## Supporting information

This article contains supporting information (7, 15, 16, 72, 73, 74, 75, 76).

## Conflict of interest

The authors declare that they have no conflicts of interest with the contents of this article.

## Supporting information

Supporting Documents S1

Supporting Documents S2
