# Supplementary material for: Aim18p and Aim46p are chalcone isomerase domain–containing mitochondrial hemoproteins in Saccharomyces cerevisiae
Source: J Biol Chem. 2023 Feb 4;299(3):102981. doi: 10.1016/j.jbc.2023.102981 (PMC9996372; doi:10.1016/j.jbc.2023.102981)
Supplement: Supporting Documents S2 [file mmc2.pdf]

Supporting Documents S2

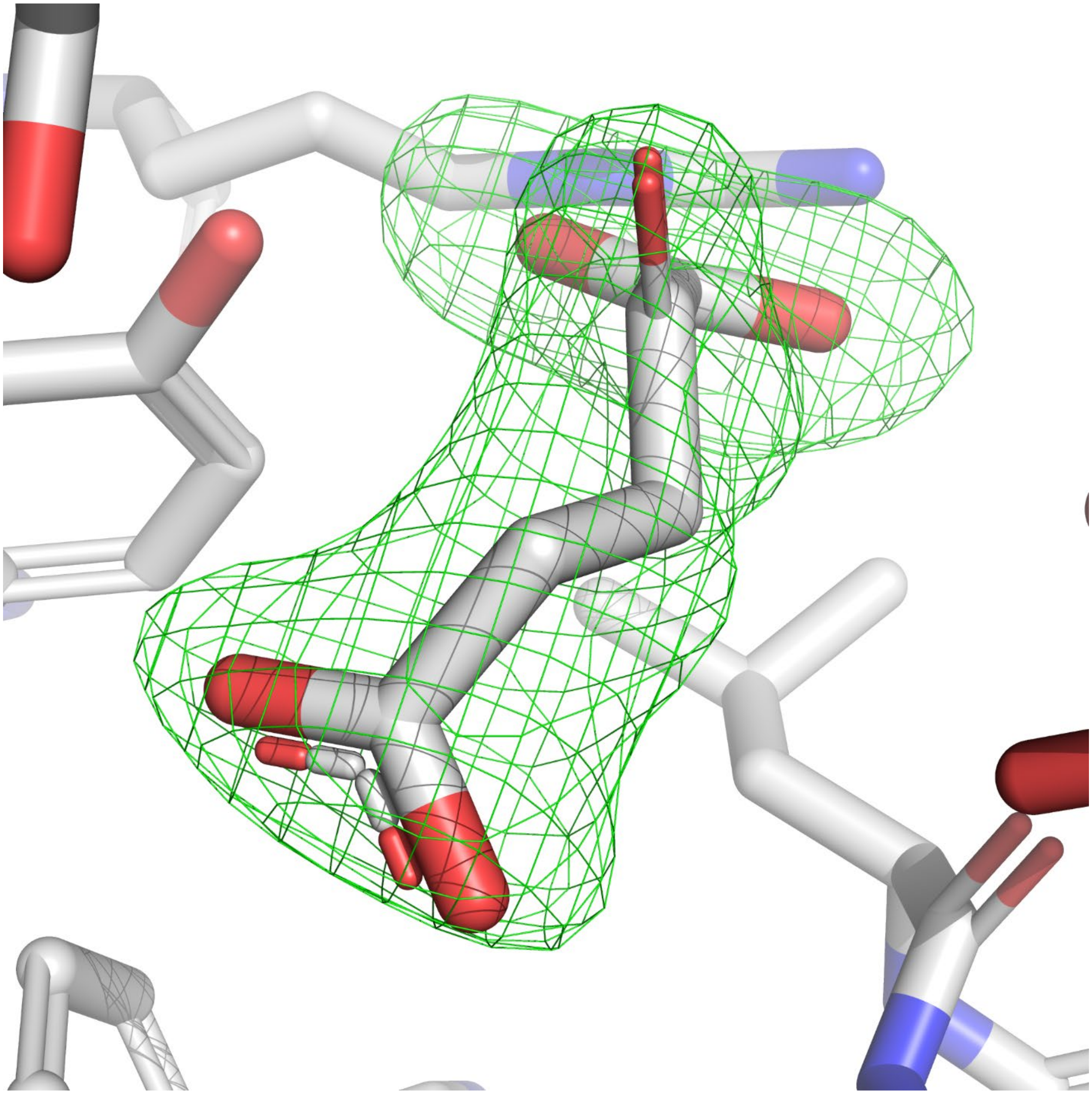

Supporting Documents S2A. mFo-Dfc difference map contoured at +3 sigma (green) with AKG omitted.

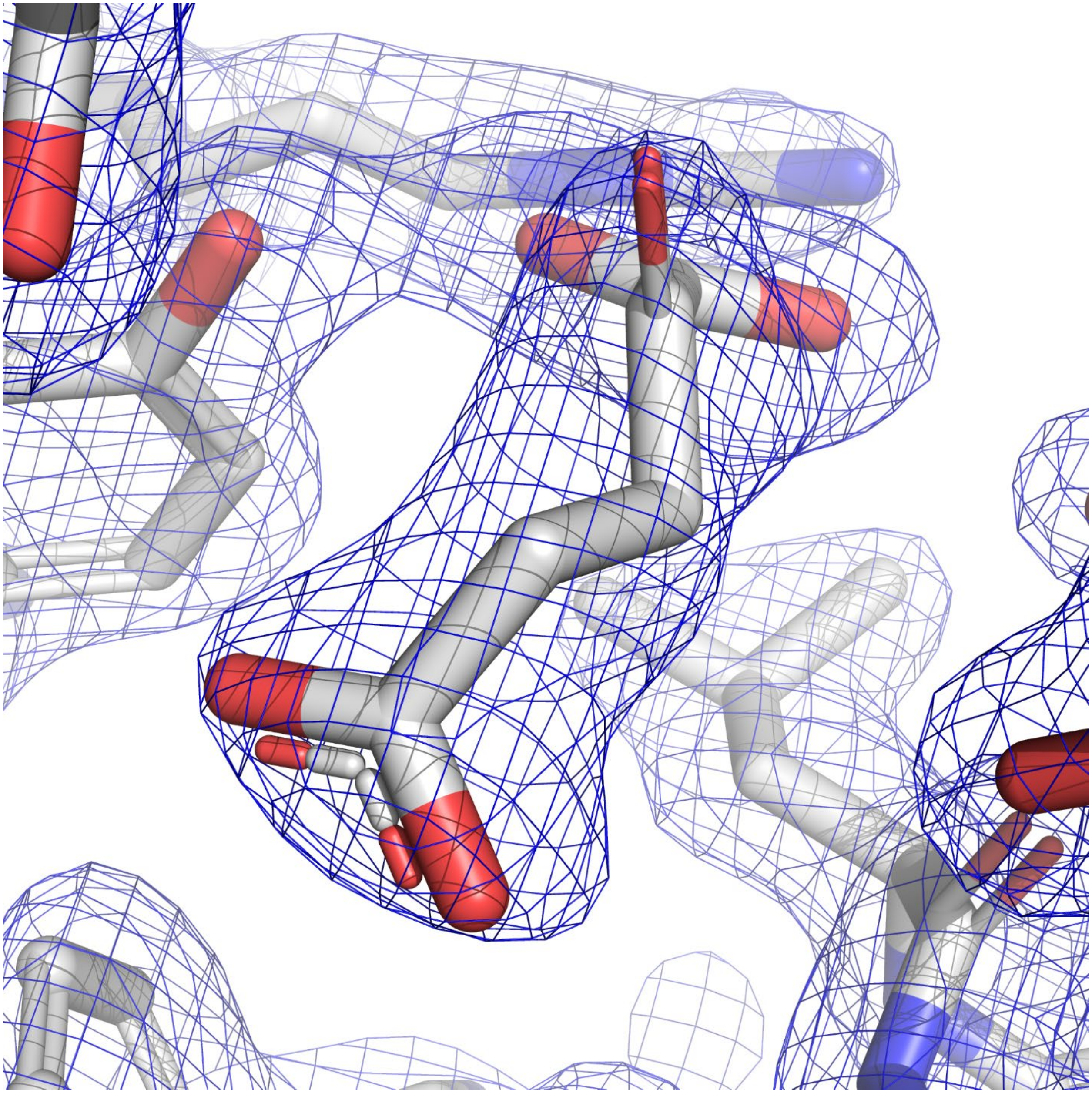

Supporting Documents S2B. Final m2Fo-Dfc map contoured at +1 sigma (blue).

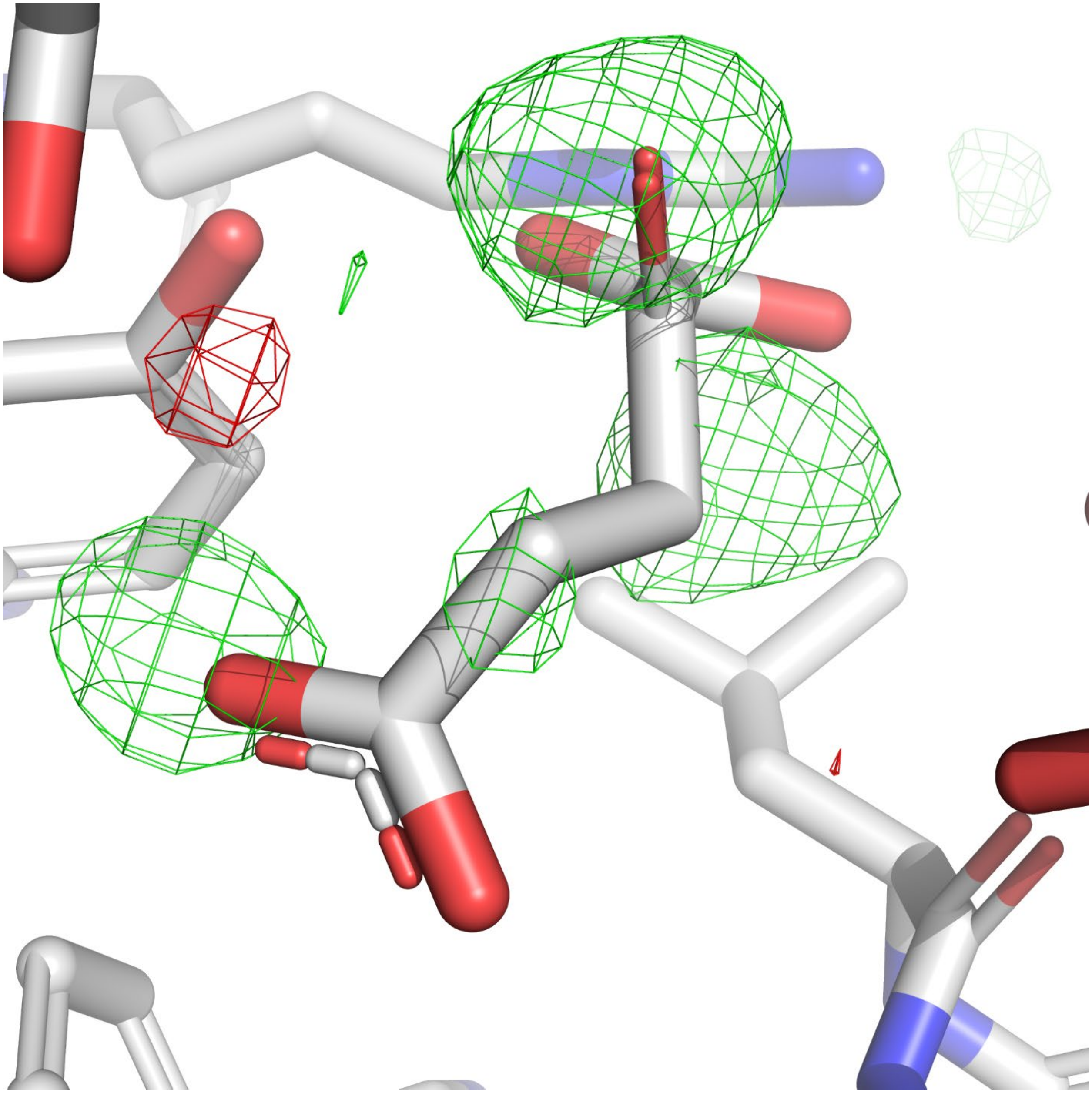

Supporting Documents S2C. Final mFo-Fc map contoured at +3 (green) and -3 (red) sigma.
